# DETECT: A MATLAB Toolbox for Event Detection and Identification in Time Series, with Applications to Artifact Detection in EEG Signals

**DOI:** 10.1371/journal.pone.0062944

**Published:** 2013-04-24

**Authors:** Vernon Lawhern, W. David Hairston, Kay Robbins

**Affiliations:** 1 Department of Computer Science, University of Texas-San Antonio, San Antonio, Texas, United States of America; 2 Human Research and Engineering Directorate, US Army Research Laboratory, Aberdeen Proving Ground, Maryland, United States of America; McGill University, Canada

## Abstract

Recent advances in sensor and recording technology have allowed scientists to acquire very large time-series datasets. Researchers often analyze these datasets in the context of *events*, which are intervals of time where the properties of the signal change relative to a baseline signal. We have developed DETECT, a MATLAB toolbox for detecting event time intervals in long, multi-channel time series. Our primary goal is to produce a toolbox that is simple for researchers to use, allowing them to quickly train a model on multiple classes of events, assess the accuracy of the model, and determine how closely the results agree with their own manual identification of events without requiring extensive programming knowledge or machine learning experience. As an illustration, we discuss application of the DETECT toolbox for detecting signal artifacts found in continuous multi-channel EEG recordings and show the functionality of the tools found in the toolbox. We also discuss the application of DETECT for identifying irregular heartbeat waveforms found in electrocardiogram (ECG) data as an additional illustration.

## Introduction

The widespread use of portable, high-throughput sensors has dramatically increased the challenges of analyzing time series data, making automated methods for time series analysis not only beneficial but necessary for practical data analysis. One area of particular interest in automated time series analysis is *event detection*. In this context, we define an *event* as an interval of time where the properties of the signal have changed relative to a baseline signal.

In this paper we describe DETECT (DETection of Events in Continuous Time), a MATLAB™ toolbox for detecting and identifying events that occur in long, multi-channel time-series. Our motivating example in developing DETECT is the problem of artifact detection in long-term EEG (electroencephalogram) recordings of brain activity. Artifacts in EEG recordings can be generated from external sources, such as power lines or electrical transformers, or from internal sources such as muscle movements like eye blinks, jaw movements or muscle twitches [1], [2]. EEG artifacts often (but not always) have signal strengths several times greater than the underlying EEG and have time scales ranging from a few milliseconds to several seconds, necessitating their removal prior to many types of analyses. Although they are most commonly considered a nuisance, some types of EEG “artifacts” such as saccades and other eye movements are intrinsic to specific types of subject behavior and may, in fact, be a variable of interest to a researcher [3]. Other applications of event-monitoring in long EEG recordings include the monitoring of epilepsy patients for epileptic seizures [4], monitoring the brain activity of newborn infants [5] as well as the monitoring of fatigue in prolonged driving simulations [6]. In many of these settings, the experiments may last several hours or days, making manual identification of events time-consuming and impractical.

Many approaches to event detection rely on using training data known to have no events. For example, [7] uses training datasets known to contain only baseline signal and calculates a metric called the importance, which is the ratio of training set to testing set densities. They estimate the importance using unconstrained Least Squares Importance Fitting (uLSIF). Methods such as a One-class Support Vector Machine (OSVM) [8] also rely on the availability of baseline training data to classify unlabeled data into two regions, baseline and events. For more dynamic data streams, DBOD-DS (Distance Based Outlier Detection in Data Streams) continuously re-estimates probability density, weighting recent points more heavily [9]. This method evaluates whether particular points are events based on this continuously evolving probability density function. Similarly, ChangeFinder [10] uses autoregressive (AR) learning together with probability densities to find events. Other approaches for event detection assume the availability of baseline training data and apply machine learning techniques to detect events [11].

Note that all of the methods described above assume the existence of training data that clearly describes the baseline, and do not provide machinery to distinguish among different types of events. This is due in part to the fact that the use of machine learning approaches for classifying different event types requires training data for each event type; however, obtaining accurate, labeled training data for real signals can be difficult and cumbersome, preventing many researchers from applying these techniques routinely in a laboratory setting. The goal of this paper is to introduce a toolbox that is simple for scientists to use, allowing them to quickly train a model on multiple classes of events, assess the accuracy of the model, and determine how closely the results agree with their own manual identification of events. The toolbox supports an iterative workflow, allowing the user to provide additional training examples or try a different feature set for creating the model in order to improve classification performance if deemed necessary. DETECT reports time indices where events occur as well as the type of event detected, and can be used to label continuous EEG data and other time series data with any event type of interest.

Here, we illustrate the use of DETECT by classifying multiple types of artifacts present in EEG recordings. Our previous work has shown that a variety of subject-generated artifacts, such as eye blinks and muscle movements, can be accurately identified within EEG by using coefficients from an autoregressive (AR) model as features for input to a support vector machine (SVM) classifier [12]. MATLAB code for the DETECT Toolbox can be found at http://github.com/VisLab/DETECT as well as on the UTSA Visualization and Modeling Laboratory (VML) website at http://visual.cs.utsa.edu/detect. Sample datasets can also be found at VML.

## Materials and Methods

### DETECT Processing Pipeline

**Figure 1:**
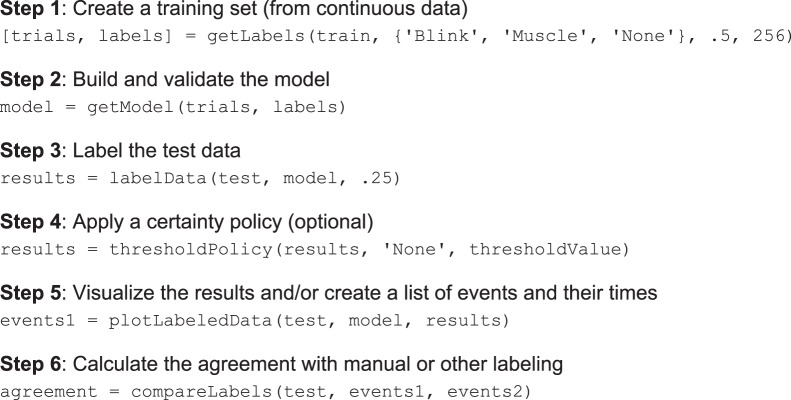
An example of the processing pipeline for the DETECT toolbox using the default settings. Step 1 creates a set of trials from training data using a window size of 0.5 s at data sampling frequency of 256 Hz. Step 2 builds and validates the model using the default settings. Step 3 labels test data using a slide width of 0.25 s. Step 4 applies an optional certainty policy based on a specified certainty threshold to relabel. Step 5 visualizes the results and produces an event log. Step 6 calculates the amount of agreement between the current labeling (events1) and another labeling (events2). Additional resources for optional parameters can be found in the DETECT Users Guide bundled together with the toolbox. HTML documentation for each function can be found at http://visual.cs.utsa.edu/detect/documentation/help/.

The DETECT processing pipeline consists of the following steps: Training set creation, model building and validation, event labeling, event visualization and assessment. Figure 1 shows an example of the DETECT function calls for each of these steps, which are explained in more detail below. These function calls describe a typical workflow using all the DETECT default parameter settings. A complete description of the function parameters and an extensive set of examples illustrating various use cases are available in the DETECT user manual distributed with the source code.

### Step 1: Create a Training Set

We assume that the data is provided as a continuous time series of multichannel data in a channels × frames array, where the columns of this matrix correspond to the time points at which the data is recorded. That is, at each time point (frame), simultaneous measurements are made from the channels and recorded. The input to DETECT model building is a three-dimensional matrix of size channels × windowSize × windows that contains the training data. The channels dimension denotes the number of simultaneous time series that are observed, windowSize denotes the length in frames of a time interval containing an event, and windows is the total number of windows (events). This array can be created manually by the user, or with the GUI described below.

**Figure 2:**
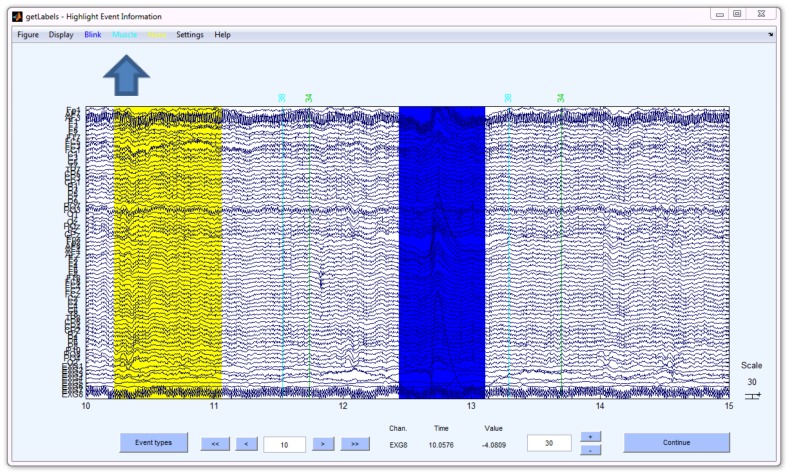
The getLabels function displays a graphical user interface (GUI) that allows users to label different events in a continuous dataset. The toolbar buttons at the top (arrow) allow the user to choose the event label. In this figure two events have been identified – a “None” event shown in yellow and a “Blink” event shown in blue.

DETECT provides the getLabels function to assist users in creating labeled features from continuous data. The getLabels function, based on EEGLAB plotting functions (Delorme and Makeig, 2004), displays a graphical user interface (GUI) that allows users to highlight and color code segments of data representing labeled events. The example GUI shown in Figure 2 uses getLabels to label artifacts in EEG. In this case, three buttons on the menu bar (see arrow) specify the allowed event labels: Blink (dark blue), Muscle (aqua), and None (yellow). The arguments of getLabels include allowed events (and colors – not shown), the desired duration of the events in seconds, and the sampling rate. In the figure, two events have been labeled: a region of no artifact, highlighted in yellow, and a region of eye blink, highlighted in blue.

To create a labeled segment, the user presses the button of the desired event type and then highlights a region of the signal by holding down the mouse button while dragging the mouse. Although DETECT downstream processing makes no assumptions about whether the training features overlap, the getLabels function does not allow overlap in labeled segments. To label a different type of event, the user simply clicks the appropriate button before highlighting. Once the user has highlighted the training data, DETECT adjusts the highlighted windows to be the same length as required by DETECT downstream processing. The window length for this example is 0.5 s, so DETECT automatically sets each data segment to be 0.5 s when the user finishes. For accurate model estimation, the number of observations in each event class should be roughly the same; DETECT issues a warning if the event classes are not balanced. There are two outputs of the getLabels function: a structure with data of size channels × windowSize × windows, and a string vector labels of length windows that contains the category for each data window. These outputs are used for model training in the next step.

### Step 2: Build and Validate a Classification Model

After producing training data, the user calls the getModel function to build the classification model for the labeled raw features. DETECT model building transforms raw features using a feature extraction function and then trains a support vector machine (SVM) classifier [13]. The feature extraction function takes an array of data (channels × windowSize × windows) and produces an array of (windows × featureSize) extracted features. A simple example of feature extraction is the standard deviation for each channel:

function features = getSTDfeatures(data)

features = squeeze(std(data, 0, 2)′);

end

The user can easily supply a feature function that is appropriate for a particular application. By default DETECT uses the getARfeatures function, which computes the coefficients of an autoregressive (AR) model of order *p* on each channel individually. The AR model of order *p* for a zero-mean time series 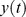 can be written as:


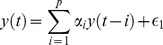


where *p* denotes the number of previous time points used to model the current time point, and *?_t_* denotes a zero-mean process with variance *σ* ^2^. We compute the AR coefficients *α_i_*, *i*  = 1, …, *p* for each channel separately and concatenate the results to form a feature vector for a window. Because each channel is treated individually, spatio-temporal information that exists in the time series cannot be directly estimated. However, the user has the option of using a different feature extraction function to extract spatio-temporal information, such as a multivariate autoregressive (MVAR) function. In fact, any feature extraction function can be used with DETECT as long as the output of this function is a matrix of size (windows × featureSize).

The getModel function uses a support vector machine (SVM) classifier for discriminating among the events based on the given features. The software library LibSVM [13] is used for fitting the SVM classifier. The getModel function uses the radial basis function (RBF) kernel by default, as this kernel has been successfully used in other classification problems [12]. The RBF kernel has two parameters: *C*, a cost penalty parameter and 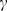 the variance parameter of the RBF [12]. The getModel function uses a grid search over 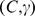 to find the pair that maximizes the cross-validation accuracy. The default grid search space is set to be large (2^[-5,10]^ × 2^[-5,10]^ in steps of .5) to account for the majority of parameter values that could arise depending on the features used. The user can modify the getModel function to increase or decrease this range if needed. The getModel function uses 10-fold cross validation by default, although this parameter can be changed by the user at the command line. The getModel function produces the estimated parameters of the SVM classifier as well as the cross-validation (CV) accuracy for the given feature extraction function. The CV accuracy can be used as a basis of comparison for validating different feature sets with two or more feature extraction functions.

### Step 3: Label the Test Data

**Figure 3:**
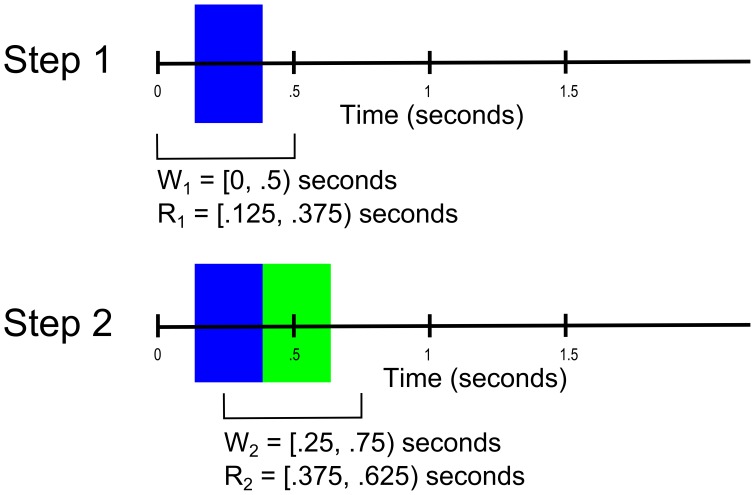
An example of the continuous detection for a window size of 0.5 s and slide width of 0.25 s. In Step 1, the analysis window W_1_ is [0, 0.5) seconds, and the prediction for this data is made at [M_1_ − 0.5 S, M_1_+0.5 S) where M_1_ =  the midpoint of W_1_ and S = the slide width. The blue color denotes the time interval of applicability of the prediction based on the first window, W_1_. In Step 2, the window is shifted by 0.25 s, so the analysis window W_2_ is [0.25, 0.75) seconds, and the prediction for the data is made at [M_2_ − 0.5 S, M_2_+0.5 S). The green color denotes the time interval of applicability of the prediction based on the first window, W_2_.

After building a classification model, the user can use the model to classify either continuous data or windowed data. The labelData function classifies continuous data using a sliding window approach and also reports the classification accuracy when the true labels are provided. Figure 3 shows the details of the sliding window.

The first region W_1_ of Figure 3 starts at time 0, and the length of the window is the width of the training windows used to build the model (0.5 s). The labelData function calculates the features in this window and uses the SVM classifier to make a prediction of the class label. The data is labeled in time using the following formula:
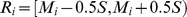
where R*_i_* is the *i^th^* region of the data, M*_i_* is the midpoint of the *i^th^* window, and *S* is the slide width, all in seconds. The prediction region, R_1_, for this label is depicted as a blue box in Figure 3. The window is then shifted in time by a user-definable slide width (in this case 0.25 s) and the process is repeated. The prediction label for the second slide is depicted by the green box in Figure 3. Data at the end of the dataset is discarded if the full time slide cannot be performed For example, if only 0.1 s of data remains at the end of the record but the slide width is 0.2 s, the remaining 0.1 s of data is not analyzed. Notice also that a time period at the beginning of the data set corresponding to ¼ of the slide width is not labeled. For data that is already pre-windowed (“epoched”), the labelWindows function classifies and reports the predicted label for each window.

### Step 4: Apply a Certainty Policy (Optional)

Both labelData and labelWindows return an additional measure of accuracy, which we call the *certainty score*. The certainty score is defined as:



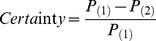
where 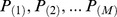 are the sorted (descending) probabilities for the *M* categories in that window as returned by LIBSVM. That is, 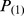 is the probability of the most likely class label for this window. If the probability distribution is mostly concentrated in one category (indicating a strong likelihood for that category), the certainty measure will be close to 1, while if the probability distribution is more evenly distributed across all the categories, the certainty measure will be close to 0. Thus this measure can be used to reveal predictions that have a high certainty of being accurate, or alternatively predictions for which the output decision is acknowledged to be potentially incorrect due to low certainty, through use of a certainty policy.

The DETECT toolbox provides two functions for implementing different certainty post-processing policies, but users can easily write additional ones to fit their criteria. These policies are implemented as functions that take either the output of labelData or labelWindows as an argument and produce a new labeling. Note that by default, the model ignores the certainty and produces a label, regardless of certainty. The example thresholdPolicy function relabels frames based on whether the certainty is greater than a user-defined threshold. If a prediction is made with a certainty that is less than the threshold, the thresholdPolicy determines whether either of the two most probable classes is the “baseline” class. If one of the classes is the baseline class, the prediction is set to be the baseline class. The prediction is not changed if either the certainty value is low but neither of the two most probable classes is the baseline class or if the certainty is above the specified threshold. The optimum certainty threshold can be found by performing a grid search along the possible values of the certainty in [0, 1].

Another sample DETECT certainty post-processing policy function, unknownPolicy, also uses a threshold, but looks at the type of the most probable event when the certainty is below the threshold. If the most probable event is the “baseline”, then the frame is labeled as the baseline. If the most probable event is a non-baseline event, then the frame is labeled as “Unknown”. Additional certainty policies can be written that use the estimated probability distribution of the classes directly, as that is one of the additional outputs from either labelData or labelWindows. Users can also write certainty post-processing policies that take into account neighboring labels in the continuous case. For example, suppose a particular event label appears as the second most likely event during one window, but neighboring windows identify the same event with high certainty. A certainty post-processing policy could relabel the window in question to account for the behavior of neighboring slides. This type of relabeling is particularly relevant for events with known physical limits on their time duration. However, it should be noted that policies will be very problem-specific, and thus certainty post-processing policies must be considered as a separate, optional step in the workflow.

### Step 5: Visualize and/or Create an Event List

DETECT has visualization tools that can be used after applying the classification model to testing data. The plotLabeledData (continuous data) or the plotWindowData (windowed data) overlay the labeling on the original signal, allowing users to evaluate the quality of the labeling or to visually compare two labelings side by side. Both functions are built on EEGLAB [14] and show a display similar to that of getLabels, illustrated in Figure 2.

### Step 6: Assess Agreement (Optional)

The compareLabels function computes the agreement between two different labelings of the same set of continuous data. Multiple labelings may occur from applying different feature extraction approaches, when using different slide parameters, or when comparing the results of the algorithm to that of manual labeling or “ground truth”. The compareLabels function uses a “fuzzy window” approach to evaluate the agreement of two labelings in time. The user sets a threshold of how many seconds of temporal tolerance are allowed when determining the amount of agreement to compensate for small differences in the exact onset and offset times that are likely to occur when applying different labeling approaches. The compareLabels treats the first labeling as “ground truth” when calculating the agreement.

A fuzzy threshold of 0 s designates no timing tolerance. In this case if both labelings designate a region as the same type of event, compareLabels assigns the region to be an Agreement. If both labelings detect a region as “baseline”, compareLabels assigns the region to be a NullAgreement. If the first (ground truth) labeling designates a region as “baseline”, but the second labeling designates the region as an event, compareLabels assigns the region to be FalsePositive. Similarly, if the first (ground truth) labeling designates a region as event, but the second labeling designates the region as “baseline”, compareLabels assigns the region to be FalseNegative. Finally, a TypeError occurs when both labelings find the region contains an event, but designate different types.

**Figure 4:**
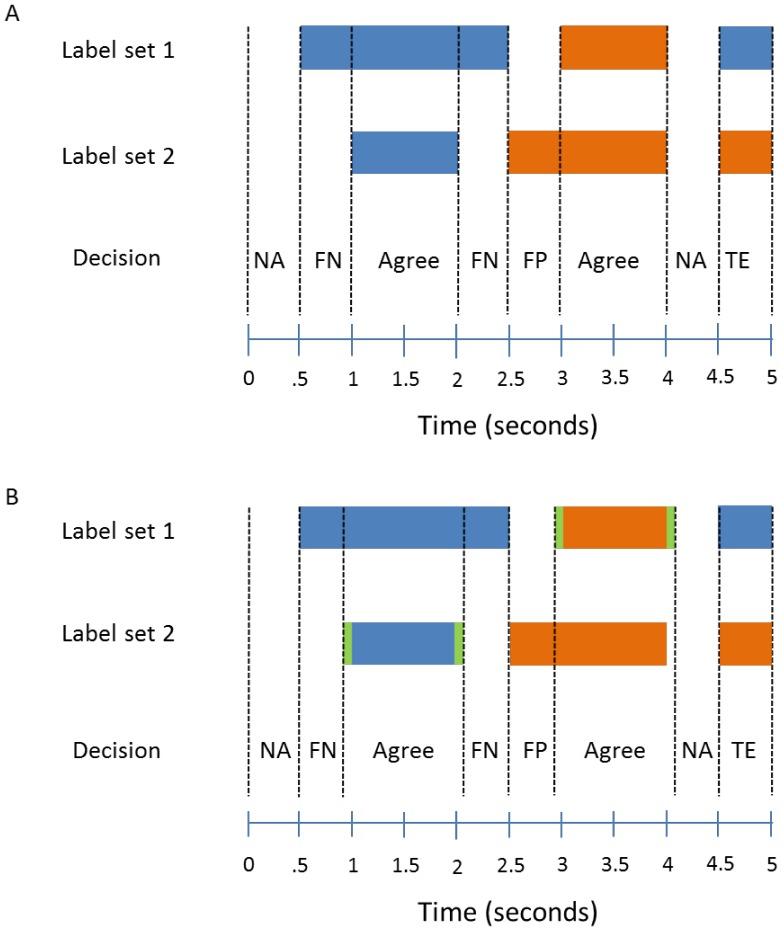
A comparison of two labeled datasets using the fuzzy window approach for allowable timing errors for two slightly different window sizes using the compareLabels function. There are two events, colored blue and orange. Blank spaces denote the absence of an event in the time window. The Decision row indicates the decision made by compareLabels. (B) The comparison with using a fuzzy window size of 100 ms. The fuzzy window extends regions of agreement by 100 ms on each end. The extension is shown in Green. The decision codes are: NullAgreement (NA), False Negative (FN), Agreement (Agree), False Positive (FP) and TypeError (TE).

Figure 4A shows an example of two different labelings of a set of frames using a fuzzy threshold of 0 s. The set has two event types, colored blue and orange. Baseline events are the periods where no events are present (in this figure, where no colors are present). In the very first period [0, 0.5]s, both labeling agree that there is no event present. This generates a NullAgreement decision (NA). In the next period [0.5, 1]s we see that Label Set 1 indicates a blue event, while Label Set 2 does not. Since compareLabels compares Label Set 2 to Label Set 1 (setting Label Set 1 to be the ground truth), this generates a FalseNegative decision (FN). In the period from [1], [2]s both labeled sets agree on the type and duration, generating an Agreement decision (Agree) for this time period. In the time region [2.5, 3]s a region is labeled in Label Set 2 and not in Label Set 1. This generates a FalsePositive decision (FP). Lastly, the region from [4.5, 5]s denotes a TypeError (TE) where the types in the two sets are not the same. After the comparison is complete, compareLabels summarizes the comparison by reporting the total time in each of the five categories.

Figure 4B shows the results of the same comparison using a “fuzzy window” of size 100 ms, which is shown in green. As before, intervals of agreement are computed by intersection. Each interval of agreement is extended on each end by the time designated by the fuzzy window parameter (in seconds). The fuzzy window parameter only applies to regions where there was an agreement between the two labelings (regions of TypeError are unaffected by this procedure). For example, the first blue region in Label Set 2 is extended on both ends by the fuzzy window, and the agreement is calculated based on this new extended time range. Similarly, the orange region in Label Set 1 is extended by the fuzzy window and the agreement is calculated based on the new range of values. The remaining conditions are then recalculated after this adjustment (FalseNegative, FalsePositive and NullAgreement).

The fuzzy window parameter can be interpreted as the allowable timing difference between two identified time regions that have the same type. It is possible that two different labelings of a dataset, while both accurate in detecting the event, are offset by a small amount in time due to the resolution of the labeling method. For example, a labeled set using one type of feature might be the result of very liberal manual labeling, while another labeled used more stringent requirements for the onset of events, resulting in slightly smaller windows in time. The fuzzy window parameter can allow the user to adjust for minor differences in the timing among two periods for determining similarity and accuracy, without being confounded by small periods of false positives or misses. Note that this approach is utilized when there is an interval of type agreement between the two labelings; no fuzzy windowing is performed otherwise. The comparison is also symmetric for regions of NullAgreement, Agreement and TypeError, but not for FalsePositive and FalseNegative decisions. In those cases, the labeling will be reversed depending on which dataset is used as the reference. In other words, FalsePositive decisions will be labeled as FalseNegative decisions when the order of comparison is reversed. The start and end time of the decisions, however, remain symmetric across all evaluation decision types.

### Toolbox Function Summary

Table 1 lists the functions found in DETECT with a brief summary of the usage. Functions are divided into two groups: functions that can be applied to any time series directly, and functions that have EEGLAB [14] dependencies. DETECT uses EEGLAB for data processing functions (such as windowing the data) as well as visualization. All functions that have plot elements have EEGLAB dependencies, which the user is required to install before using DETECT. Details about the parameters needed for each function can be found at our HTML help page at http://visual.cs.utsa.edu/detect/documentation/help/.

**Table 1:** DETECT Function List and Summary.

GENERAL FUNCTIONS (can be used to label any type of time series data)	
Name	Description
getARfeatures	Estimate autoregressive model coefficients of specified order for a 3D array of input data (*channels* × *windowSize* × *windows*) and return a (*windows* × *featureSize*) array of features to be used for classification.
getModel	Create a model or classifier based on data (*channels* × *windowSize* × *windows*) array of training data and a (*windows* length) vector of class labels.
labelData	Label data (*channels* × *frames*) as a function of time based on a classification model and report certainty of each label.
labelWindows	Label windows (*channels* × *windowSize* × *windows*) based on a classification model and also return classification accuracy if ground truth labels are passed in for comparison.
compareLabels	Compares two sets of labeled data, either from an automated labeling (by DETECT) or from manual labeling (using markEvents) or both (one set from a manual labeling and the other from an automated labeling).
thresholdPolicy	Applies the threshold post-processing certainty policy used in this paper. If the certainty is below a given threshold and one of the top two classes is the baseline, the prediction type is set to the baseline. No change is made if neither of the top two classes is the baseline.
unknownPolicy	A post-processing certainty policy that incorporates a new decision class of “Unknown”. If the certainty is below a given threshold and one of the top two classes is the baseline, the prediction type is set to the baseline. Otherwise, the prediction type is set to “Unknown.”
**EEG RELATED FUNCTIONS (depend on EEGLAB)**	
getLabels	Convert a continuous dataset into an epoched or windowed dataset, epoching by user-highlighted regions.
plotLabeledData	Display results of continuous labeled data using a modified EEGLAB plot window.
plotMarkedData	Plot a manually labeled dataset using a modified EEGLAB plot window.
plotWindowData	Display results of labeling windowed dataset using a modified EEGLAB plot window.
markEvents	Manually label data based on given categories. Can update a previously labeled dataset to add/remove events and categories. Uses a modified EEGLAB plot window.

Additional information about the optional parameters and their default settings can be found by viewing the HTML function help pages located at http://visual.cs.utsa.edu/detect/documentation/help/. Additional information can also be found in the DETECT Users Guide, provided with the toolbox.

### Experimental Setup

Here we highlight the utility of DETECT using the problem of artifact detection in EEG. Data was acquired from three participants who performed a standard visual-evoked-potential (VEP) task, in which they reported whether each presentation of an image was a depiction of a U.S. Soldier (common) or an enemy insurgent (infrequent). All participants provided written consent prior to participating, and methods were approved as required by U.S. Army human use regulations [15], [16]. The EEG data was recorded at 512 Hz using a 64-channel Biosemi ActiveTwo system (Biosemi, Amsterdam, Netherlands) and referenced to the average of the two mastoids. Four external channels were used to record eye movements by electrooculography (EOG). EOG activity was recorded to verify the instances of eye blinks and saccades in EEG, but this information was not used in subsequent analyses. The data were down-sampled to 256 Hz using a discrete wavelet transform from the Meyer wavelet family. The approximation coefficients of the down-sampled signal were then high-pass filtered at 1 Hz using an order 8 IIR Butterworth filter. We used EEGLAB [14] for processing and ERPLAB [17] for filtering the data. A total of three datasets, labeled D1, D2 and D3, were used for this study. The three data sets differed significantly in their event content: the first dataset (D1) was fairly clean with few artifacts, while the remaining two datasets (D2 and D3) had significantly more artifact contamination as well as more complex combinations of events.

Three users, each with more than 10 years of EEG experience, were asked to label the three datasets using the markEvents function in DETECT (see Table 1). This function generates an interactive GUI that can be used to label continuous data efficiently with different classes of events. The users categorized events into three classes: Blink, Eye and Muscle. ‘Blink’ denotes eye blinks in the EEG signals, ‘Eye’ denotes eye movements such as vertical or horizontal saccades, and ‘Muscle’ denotes periods of extensive high-frequency muscle activity. The users were not given any specific instructions about labeling, and were asked to apply their individual working criteria for identifying artifacts. They did not discuss their criteria with each other prior to the labeling. Due to the fairly low frequency of muscle and eye-movement artifacts (and a high frequency of blink artifacts) present in each of the subjects data, we used data acquired from the same subjects that was previously labeled for artifacts as the training data for building an artifact discrimination model [12]. In this dataset, subjects were instructed to make specific artifact-inducing movements that commonly contaminate EEG signals, such as eye blinks and jaw movements. Using this artifact battery, an artifact discrimination model was built uniquely for each subject. Our previous research [12] showed that an order 2 AR model maximized the cross-validation accuracy for distinguishing among the artifact conditions. Therefore, we used an order 2 AR model as the feature extraction function for all analyses presented throughout the paper.

## Results

### Comparison with Manual Labeling by Users

Agreement between the user evaluation and the results of DETECT were determined by using the fuzzy window method for comparing labeled regions. We designated the user labeling as the reference data set in comparisons with the compareLabels function, with a slide width of 125 ms for labeling the data, as it gave good timing localization of artifacts while still being computationally efficient. When comparing the user-labeled data with the automated labeling by DETECT, we used a fuzzy window parameter of 100 ms, allowing for 100 ms of tolerance on either side of the window. These comparisons demonstrate the software use, while illustrating some of the issues that arise in labeling such data. The datasets from the three participants are labeled D1, D2 and D3 in the following discussion. Although these sets were acquired under the same experimental conditions, their artifact content differed significantly. D1 has relatively few, well-defined artifact events, while D2 and D3 have more complex events where different types are sometimes intermixed.

Table 2 shows the results when comparing the labeling for dataset D1 to the automated labeling by DETECT. We see that there is significant agreement across all three users, with over 97% agreement in all cases. This accuracy percentage was obtained by combining the decisions Agreement and NullAgreement, as the absence of artifacts in both datasets constitutes an agreement.

**Table 2:** Summary of the comparison between different user labelings and automated labelings by DETECT for Dataset D1.

	Total Agreement	Agreement	Null Agreement	Type Error	False Positive	False Negative
User 1	469.488 s (97.81%)	14.109 s (2.94%)	455.339 s (94.7%)	2.559 s (0.53%)	1.680 s (0.35%)	5.902 s (1.23%)
User 2	472.805 s (98.5%)	14.523 s (3.03%)	458.282 s (95.53%)	2.141 s (0.45%)	1.559 s (0.32%)	3.227 s (0.67%)
User 3	474.422 s (98.84%)	14.176 s (2.95%)	460.246 s (95.95%)	1.684 s (0.35%)	1.551 s (0.32%)	2.055 s (0.43%)

Agreement between user and DETECT labeling is measured in total seconds of the data for dataset D1. The percentages denote the percent of the data that was in each of the five comparison categories. The Total Agreement column is the sum of the Agreement and Null Agreement columns. A certainty threshold value of 0.5 was used with the thresholdPolicy post-processing function.

**Figure 5:**
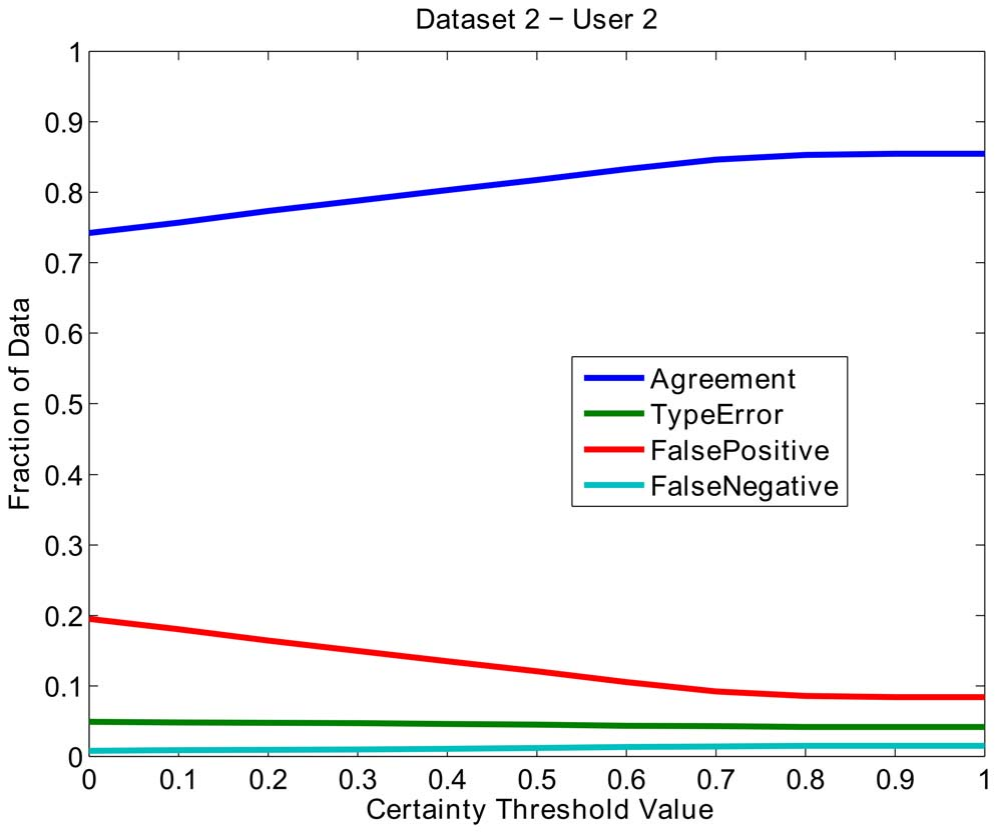
Fraction of the data in different groups when using different threshold values in the thresholdPolicy certainty policy (Agreement and NullAgreement are combined).

We used the thresholdPolicy post-processing policy function to remove false positives in the data. The optimal threshold parameter was estimated by maximizing the summed values of Agreement and NullAgreement over a grid search of possible threshold parameters. Our grid is from [0, 1] in increments of 0.1. For Dataset D1, this value was estimated to be 0.5. An example of this procedure is shown in Figure 5 for Dataset D2. A threshold value of 0 means there is no certainty policy applied to the predictions, and any predictions made with low confidence are included in the analysis. The Agreement score increases (and the corresponding FalsePositive rate decreases) as the threshold increases from 0 to a plateau of approximately 0.7, after which threshold increases have little effect. TypeError and FalseNegative rates do not have a significant dependence on the threshold for this dataset.

**Figure 6:**
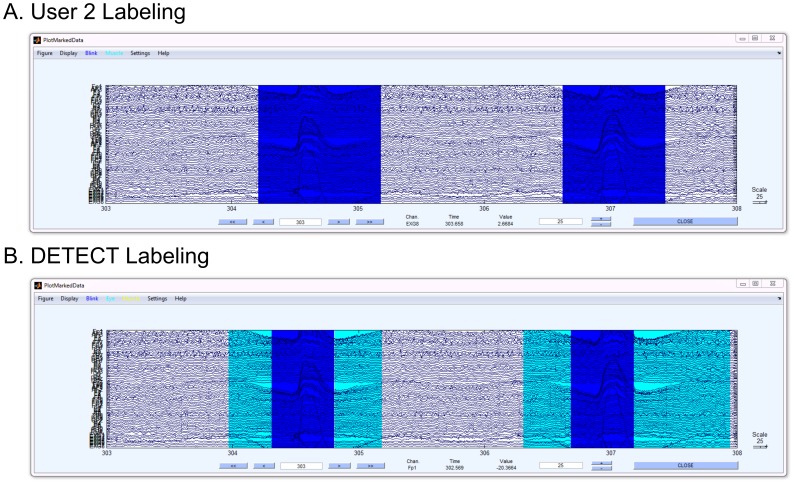
Example of disagreement between the labeling from User 1 (top graph) and DETECT (bottom graph) for dataset D2.

Figure 6 compares an example of a labeling disagreement between a series of labels by User 1 and automated DETECT labeling (bottom) for dataset D1. Both labelings recognized two distinct eye blinks, but DETECT classified regions at the beginning and end of these blinks as eye movements (aqua regions). This discrepancy can be explained by noting that DETECT makes a local decision on the basis of each window. Specifically, blinks have similar characteristics to upward eye movements at the beginning of the movement, resulting in misclassification until a sufficient amount of the signal falls within the window of analysis, after which point DETECT makes the correct labeling. In contrast, a human user is more likely to observe the entire time course simultaneously, leading to an immediate identification of the movement type based on more global information.

Table 3 shows the labeling agreement results for dataset D2. The estimated certainty threshold was found to be 1.0 when using a grid-search. The labeling agreement for D2 was not as high as for the less complex D1. For example, the results for User 1 had a higher FalseNegative rate than those for the other users, which was due to User 1 identifying wider intervals for the individual artifacts. In contrast, DETECT labeled smaller regions in time overall, even when using the fuzzy window parameter.

**Table 3:** Summary of the comparison between user labelings and automated labelings by DETECT for Dataset D2.

	Total Agreement	Agreement	Null Agreement	Type Error	False Positive	False Negative
User 1	430.973 s (82.25%)	54.172 s (10.34%)	376.801 s (71.91%)	23.621 s (4.5%)	39.270 s (7.49%)	27.648 s (5.28%)
User 2	447.809 s (85.46%)	50.879 s (9.7%)	396.930 s (75.75%)	21.891 s (4.18%)	44.109 s (8.42%)	7.934 s (1.51%)
User 3	446.492 s (85.21%)	52.371 s (9.9%)	394.121 s (75.21%)	19.867 s (3.79%)	44.238 s (8.4%)	11.000 s (2.1%)

Agreement between user and DETECT labeling is measured in total seconds of the data for dataset D2. The percentages denote the percent of the data that was in each of the five comparison categories. The Total Agreement column is the sum of the Agreement and Null Agreement columns. A certainty threshold of 1 was used.

**Figure 7:**
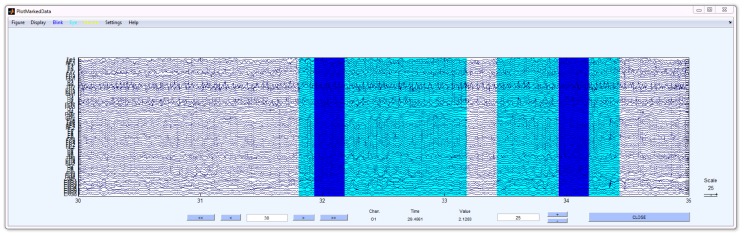
An example of a FalseNegative error in dataset D2 when using DETECT. Increased periods of alpha spindling are found in the highlighted regions of the data. These regions are being misclassified as Eye and Blink activity.

The higher FalsePositive rate for D2 was due in part to additional activities found in the EEG signal that were not labeled by the users. This dataset in particular contained periods of increased alpha activity, an example of which is illustrated in Figure 7. In this case, DETECT often recognized that this alpha activity was significantly different than the baseline signal, resulting in a FalsePositive error.

**Figure 8:**
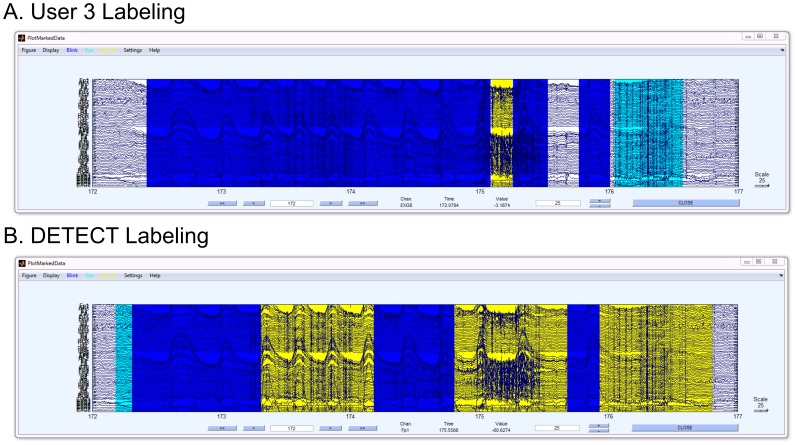
A segment of dataset D2 with regions labeled from a user (A) and the automated labeling from DETECT (B).

Dataset D2 also shows a higher TypeError rate than dataset D1, due to the difficulty of distinguishing types in complex events (such as the situation shown in Figure 8). Here, User 2 (top) labeled the entire region from about 172.5 s to 175 s as blink activity (blue), while DETECT labeled a small region in the middle (from 173.5 s to about 174.3 s) as muscle activity (yellow). The yellow region clearly has a mixture of artifact types (blinking activity and muscle activity), and the policy used for this figure selects the most likely artifact type. An alternative certainty post-processing policy could be used to report additional artifact type candidates, such as the two most likely types when there is not a clear winner as determined by the certainty measure. Table 4 shows the disagreement rates for dataset D3.

**Table 4:** Summary of the comparison between user labelings and automated labelings by DETECT for Dataset D3.

	Total Agreement	Agreement	Null Agreement	Type Error	False Positive	False Negative
User 1	478.004 s(84.6%)	106.219 s(18.8%)	371.785 s(66.13%)	17.621 s(3.12%)	38.980 s(6.9%)	27.965 s(4.95%)
User 2	478.031 s(84.6%)	106.531 s(18.86%)	371.500 s(65.75%)	17.621 s(3.12%)	38.859 s(6.88%)	28.059 s(4.97%)
User 3	474.770 s(84.03%)	107.012 s(18.94%)	367.758 s(65.47%)	23.531 s(4.16%)	30.367 s(5.37%)	33.891 s(6%)

Agreement between user and DETECT labeling is measured in total seconds of the data for dataset D3. The percentages denote the percent of the data that was in each of the five comparison categories. The Total Agreement column is the sum of the Agreement and Null Agreement columns. A certainty threshold of 0.8 was used.

### Computational Complexity Analysis

DETECT, due to its simple framework and construction, may have an application in identifying events in near real-time, allowing for the potential extension to Brain- Computer Interface (BCI) applications, such as monitoring during prolonged experiments.

We evaluated performance with respect to three factors: the order of the AR model used for our features, the slide width, and the number of channels used to fit the model. We used a Windows™ laptop computer with a 2.5 GHz Intel™ processor and 8 GB of memory for calculating the computational times. No hyper-threading or other parallel processing was utilized. Total computational time scales inversely with the slide width (taking half of the slide width means twice as many slides needed to analyze the same amount of data). The computational time is dominated by the cost of feature selection. We found that the computation time scales linearly with the number of channels; this is because we fit each channel separately and concatenate the AR coefficients to form the feature vector. The performance also scales linearly with the order of the AR model. The remainder of the calculation is relatively independent of the size of the features. When using approximately 9 minutes of data, having 8 channels and being sampled at 256 Hz, the total computational time is reduced to about 6 seconds. Note that this time could be somewhat reduced by multi-thread computing with each channel analyzed in parallel or by optimizing the linear regression algorithms for the AR model computation.

### Heartbeat Abnormality Detection using DETECT

To show the applicability of the DETECT toolbox to other time series data, we conducted an analysis of electrocardiogram (ECG) data, which was obtained freely from the online PhysioNet database [18]. For our analysis, we downloaded the data in EDF format from subject 14046 from the MIT-BIH Long Term Database [18] and imported the data directly into EEGLAB. We also downloaded a list of previously marked event times for this dataset. These event times were taken as the ground truth and used for classification model building. This dataset contains two types of ECG waveform information: the normal heart waveforms and premature ventricular contraction (PVC) waveforms. The ECG data, which was approximately 24 hours in length, was sampled at 128 Hz. The goal of our analysis was to distinguish between a normal heart waveform, a PVC waveform and the absence of a heart waveform (this is treated as the baseline condition) using both ECG channels in the analysis.

**Figure 9:**
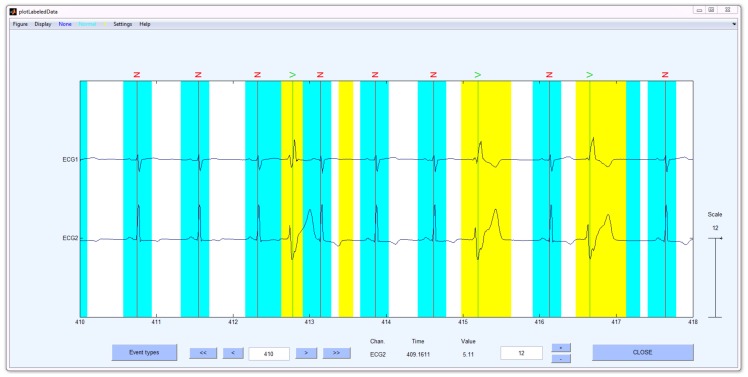
DETECT labeling of ECG data obtained from the PhysioNet online database. Aqua regions denote predictions of normal heart waveforms while yellow regions denote predictions of PVC waveforms. Periods of no color shading denote the absence of a heart waveform. The event codes N and V denote a Normal and PVC waveform based on expert labeling, respectively.

We extracted two non-overlapping sections of 10 minutes in length from the original 24 hour recording. One section was used for training the classification model, while the other section was used for testing and display purposes. The getLabels function was used on the training dataset to manually extract 20 windows for each of the three conditions (normal heart waveform, a PVC waveform, and the baseline condition containing no heartbeat). We set the window size to be 400 ms as this encapsulated the time course of the heart waveforms. For building the classification model, we used the function getModel using an order 4 autoregressive model. An order 4 model was chosen as this value maximized the cross-validation accuracy (∼98.3%). For continuous labeling, we called the labelData function with a sliding window value of 100 ms. The results of our analysis are shown in Figure 9. Here we see that DETECT was able to identify both the normal heart waveform (N) and the PVC waveform (V), and was able to accurately distinguish between the two conditions. Regions between the heart beats are correctly identified as the baseline condition (the absence of a heartbeat).

## Discussion

DETECT (DETection of Events in Continuous Time) is a MATLAB toolbox for detecting and identifying events that occur in time series data using a sliding window approach. A goal of DETECT was to provide the tools needed for scientists to easily train and test models for identifying artifacts or other events without extensive programming or machine learning experience. DETECT can handle ordinary data matrices as well as EEGLAB EEG datasets, making it a tool that can be used outside of the neuroscience community for time series applications.

A typical DETECT workflow begins with a user manually identifying a small set of regions containing events of interest, building a model, and assessing agreement. If the model does not perform sufficiently, the user continues to build the training set, often by relabeling regions of disagreement, and iterates the model training process. For example, we showed that DETECT was capable of identifying additional activities in the EEG that were sufficiently different from the baseline condition (the alpha spindling activity in Figure 7). If the user thinks this activity is a false positive, the model could be re-trained to include these activities as part of the baseline category. The user could also define a new category for this activity, re-train the model and extract other periods in the data that have the same characteristics. The process continues until the user is satisfied with the accuracy of the model. Users should always run a consistency check using compareLabels to make sure that classification accuracy on the training data is close to 100% with their own manual labeling. If this is not the case, then users should consider other feature sets.

By default DETECT uses AR features, but allows feature functions to be passed as an argument to the model builder. We showed in previous work [12] that AR coefficients formed reliable feature sets that can be used to detect artifacts in EEG recordings. AR coefficients are scale and location invariant, making these features suitable for classifying artifacts across multiple subjects. Since we omit the intercept of the AR model as a feature for classification, the AR coefficients are location invariant. Similarly, the AR coefficients are scale invariant because the signal variance is not used. Because the AR model relates the signal at the current time to its previous time points, the AR coefficients are invariant to scaling factors since the scaling factor cancels out in the estimation procedure [12]. One limitation of our approach is that, by fitting an AR model to each channel individually, some spatio-temporal information from the original data is lost. While this approach works well for identifying EEG artifacts, as shown in our previous work [12], this approach may not work well for other types of events where the events are associated with spatio-temporal characteristics. Alternative feature functions, such as multivariate autoregressive (MVAR) models which can estimate relationships both within and across channels, will be needed in these scenarios. Other features such as the mean and standard deviation of each channel can be calculated; however, these features are inherently dependent on the scale of the data and may be too sensitive to outliers to be robust measures of events, at least in EEG time series.

The DETECT framework has several other parameters that allow users to customize behavior for particular applications, such as the window size, slide length, and certainty policy. Window sizes should be set large enough to encompass entire events. If the window size is too small, then the window may not contain sufficient information to model the feature. On the other hand, if the window is too large, DETECT will not be able to distinguish closely-spaced events, and temporal precision regarding the exact onset of an event is diminished. DETECT produces a probability distribution for all class labels. Users could implement a post-processing certainty policy that outputs multiple labels if the probabilities for multiple events are comparable. A user can also apply the GUI independently for each type of artifact and then combine the resulting labels into a larger feature set. In this case there is no restriction on the fact that these epochs overlap since the model doesn’t care what time these different epochs occurred at.

DETECT uses a sliding window approach for labeling events in time and makes each prediction independently of the previous prediction. With small slide widths, the analysis windows have significant overlap; thus, the predictions may not be independent. One can implement certainty policies that take into account the time history, such as Hidden Markov Model (HMM) approaches, where the current prediction would have a high probability of being in the same class as the previous prediction if the certainty of prediction falls below a threshold level. Another possible certainty policy uses a minimum run length rule for the predictions. For example, eye blinks last on average 300–500 ms, so if the slide width is much smaller than this value (say, 50 ms) the number of consecutive predictions of the same class can be used to determine the validity of the prediction. Because certainty policies are applied after the initial model building, users can compare the accuracy and/or agreement among several certainty policies and thresholds by separately applying the policies to the output of labelData and using compareLabels for assessment.

Computationally speaking, DETECT is very efficient in labeling and identifying artifacts in EEG signals when using AR coefficients as features. In fact, we found that DETECT takes about 6 seconds to analyze 9 minutes of data at 256 Hz when using eight (8) EEG channels, suggesting that this approach can be used for near real-time detection of artifacts in EEG signals. This computational efficiency can be improved by using distributed computing to fit the AR coefficients, as each channels’ coefficients are estimated independently of the other channels. In the case of artifact detection at least, the AR features are highly redundant, and a channel subset that includes a few frontal channels for detecting eye and forehead muscle movements, and a few posterior channels to detect neck muscle activity can likely achieve comparable accuracies to the 64-channel recordings used in this paper. This observation is particularly relevant for newer wireless EEG headsets, which are bandwidth-limited and tend to have only a few channels when compared to wired systems.

The motivating example of DETECT was continuous artifact detection in EEG data, which allows the removal or preprocessing of artifact-contaminated regions. Because DETECT can label data according to different artifact types, it is possible to develop specific routines that can be used whenever certain artifacts are detected. For example, a regression-based technique for removing eye blink artifacts in EEG has been proposed in the literature [19], [20], [21]. Maximum Signal Fraction Analysis (MSFA), which is based on an eigen-decomposition of the multi-channel EEG that uses covariances and lagged autocorrelations, has been used to remove artifacts in EEG signals and is computationally efficient in short time windows [22]. However, this technique requires prior knowledge of where the artifact period is. Thus, one could use DETECT to locate target artifact time periods in the data, then use MSFA to remove them locally from the data without the need to process the entire dataset. Alternatively, DETECT can be applied in conjunction with more global artifact removal techniques such as ICA [23], [24], [25], [26] to assess the amount of signal loss in regions where no artifact was detected.

There are several approaches specifically for detecting and removing artifacts from EEG time series. SCADS [27], or Statistical Control of Artifacts in Dense Array Studies, is a method for removing artifacts in EEG before the analysis of event-related potentials (ERPs). SCADS uses a multi-pass method, where in the first pass channels and trials containing artifacts are rejected from the recording reference to prevent artifact propagation when transforming to an average reference. In the second pass, SCADS identifies and rejects trials and channels using a combination of visual inspection and summary statistics such as the standard deviation. Data from removed channels are interpolated from neighboring channels to preserve the original number of channels in the data. Note that the first pass of SCADS is fully automated, while the second pass is not. FASTER [25], or Fully Automated Statistical Thresholding for EEG Artifact Rejection, is a MATLAB Toolbox that uses a multi-stage pipeline for automated EEG artifact removal. In the initial pipeline stages, FASTER uses summary statistics, such as the variance, mean correlation and spatial kurtosis to detect bad channels in the data. FASTER then applies independent component analysis (ICA) [23], [28] to remove eye artifacts. At each stage, z-scores provide the removal criteria and removed channels are interpolated to preserve the original number of channels. Both SCADS and FASTER methods were designed primarily for the analysis of event related potentials (ERPs). These methods were also designed for detecting artifact characteristics in channels/trials, and not necessarily the identification of artifacts in the time domain, where it may be useful to know the frequency and duration of specific types of artifacts in the EEG dataset.

Some software packages that have been developed for EEG data analysis have routines aimed specifically for detecting artifacts. For example, EEGLAB [14] contains functions for detecting artifacts in EEG time series by first windowing the data into non-overlapping windows and calculating statistical summaries of the data (such as the kurtosis and standard deviation). Users then define threshold values for detecting artifacts in the now-windowed data; however, this requires prior knowledge of the appropriate threshold values, which can be challenging to resolve. Another software analysis tool, FieldTrip [29], includes functions that can identify several types of artifacts including high frequency muscle, eye activity and heart rate artifacts. These functions generally band-pass filter the data to a specific frequency range for artifact identification; for example, the eye artifact detection routine suggests using a band-pass filter range of [1], [15] Hz and a [110, 140] Hz band-pass filter range for muscle activity. Several parameters, including z-score thresholds and filter properties, are used to detect artifact regions in the data. Similar to EEGLAB, the difficulty with these routines is that the user has to manually define the parameters for detecting each artifact category. While FieldTrip provides general recommendations for parameter values for specific artifacts, scientists may need to tune the parameters for specific experiments or subject characteristics. Furthermore, it is not always clear how the parameters should be chosen for a particular dataset and how robust these parameter values are for different datasets. One key feature of DETECT is that it does not require the user to manually define threshold values for detecting events, as the thresholds are estimated by the SVM. This helps reduce some of the subjectivity in the artifact detection routine.

As a fast-detection tool, the labeled data from DETECT can also be used as an additional channel of information useful for analysis. In EEG experiments, for example, eye blink frequency and duration could be used to monitor subject performance during the task, as these features have been linked to drowsiness and fatigue [3]. Deviations from a “normal” blink frequency and blink duration distribution can be calculated in a baseline state, and measured against future data for deviations from the baseline distribution using methods of outlier/change-point detection but applied to a time series derived from DETECT output such as the inter-blink interval. DETECT could also be used for data mining purposes, where a large collection of datasets needs to be analyzed for particular events.

We have written DETECT with minimal software dependencies, only requiring the basic version of MATLAB™ without any additional MATLAB Toolboxes, and as such is functionally independent of any operating system. Bundled together with DETECT are the necessary software packages to perform AR modeling of time-series using the Time Series Analysis (TSA) Toolbox [30], as well as SVM fitting software using LibSVM [13]. DETECT, as well as its dependencies, are all free and open-source software packages released under the GNU General Public License (GPL). The DETECT plotting functions are built on EEGLAB [14], another freely available MATLAB toolbox.
